# Function‐Preserving Tailored Open Partial Laryngectomy for Chondrosarcoma of the Thyroid Ala: A Case Report

**DOI:** 10.1002/oto2.72

**Published:** 2023-09-05

**Authors:** Janice L. Farlow, Norman D. Hogikyan, Robert J. Morrison

**Affiliations:** ^1^ Department of Otolaryngology–Head and Neck Surgery Michigan Medicine Ann Arbor Michigan USA; ^2^ Department of Otolaryngology–Head and Neck Surgery Indiana University School of Medicine Indianapolis Indiana USA

**Keywords:** chondrosarcoma, function preserving, larynx, organ preservation, partial laryngectomy

Chondrosarcomas are the most common non‐squamous cell laryngeal cancer^1^ and are almost exclusively treated with surgery.^2^, ^3^ Given that most chondrosarcomas are low grade with a 5‐year survival of approximately 90%,^2^ preservation of function is key. Traditional partial laryngectomy approaches preserve a portion of the laryngeal framework but still have a significant impact on laryngeal function.^4^ Herein, we present a unique approach to function‐preserving open partial laryngectomy that fully resects involved laryngeal framework while preserving all internal laryngeal tissues, thus requiring no tracheostomy and minimal postoperative care.

## Case

**Figure 1:**
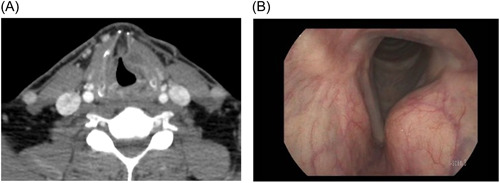
Preoperative views of neoplasm. (A) Axial section of computed tomography scans at the level of the thyroid cartilage, demonstrating well‐circumscribed homogeneous hypodense lesion within the left thyroid ala. (B) Flexible laryngoscopy demonstrating supraglottic submucosal mass.

A 54‐year‐old male with no significant cardiopulmonary comorbidities presented with a left thyroid ala chondrosarcoma (Figures 1A and B). This study was deemed exempt by the Institutional Review Board.

### Exposure of the Larynx

**Figure 2:**
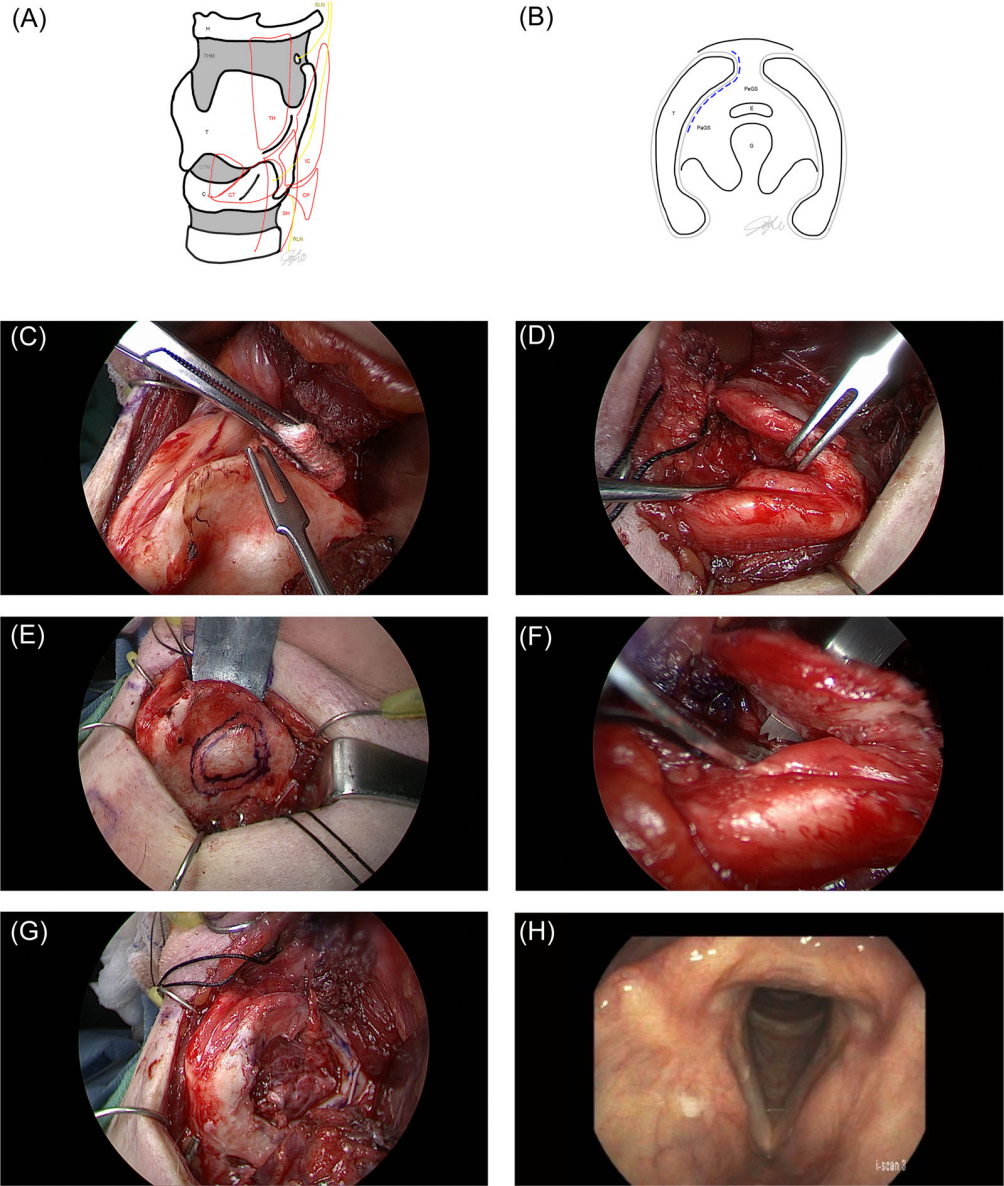
Laryngeal framework with relevant anatomy. (A) Oblique and (B) axial views at the level of the thyroid ala. The dotted line demonstrates dissection in the paraglottic space. Intraoperative (C) oblique and (D) superior views of the paraglottic space, with (E) marking and (F) resection of the neoplasm and (G) the final defect displayed. (H) Flexible laryngoscopy at 10 weeks postoperatively. C, cricoid cartilage; CP, cricopharyngeus muscle; CT, cricothyroid muscle; CTM, cricothyroid membrane; E, epiglottis; G, glottis; H, hyoid; IC, inferior constrictor muscle; PaGS, paraglottic space; PeGS, pre‐epiglottic space; RLN, recurrent laryngeal nerve; SLN, superior laryngeal nerve; ST, sternohyoid muscle; T, thyroid cartilage; TH, thyrohyoid muscle; THM, thyrohyoid membrane.

After raising subplatysmal flaps and lateralizing strap musculature, the sternohyoid muscle is divided. Muscular attachments are freed from the oblique line and the inferior border of the thyroid ala. Dissection is not carried further posterior to the oblique line or inferior to the cricothyroid joint to avoid damage to the external branch of the superior laryngeal nerve and recurrent laryngeal nerves, respectively (Figures 2A and B).

### Separation of the Laryngeal Framework

The thyrohyoid membrane is sharply incised along the superior border of the thyroid ala. Once the plane between the inner perichondrium and endolaryngeal soft tissues is established, blunt dissection is performed into the paraglottic space, separating intrinsic laryngeal contents from the thyroid ala (Figures 2C and D). Care is taken to preserve thyroid cartilage perichondrium and to avoid dissecting too inferiorly in the anterior midline where the anterior commissure attaches to the thyroid cartilage. The cricothyroid membrane is similarly freed from the inferior thyroid ala.

### Resection of Neoplasm

While a malleable retractor protects the paraglottic soft tissues, the ala is incised with an 11‐blade or an oscillating saw for areas of ossification. Once the neoplasm is freed circumferentially, it can be removed en bloc (Figures 2E‐G).

### Reconstruction and Closure

In this case, horizontal and vertical struts of the thyroid ala were preserved. Titanium plating or cartilage grafting can be considered otherwise to maintain vocal fold tension and luminal volume. Paraglottic soft tissues are re‐suspended from the edges of the defect. Inferior and superior sternothyroid muscle components can then be reapproximated. The remainder of the strap musculature can be loosely reapproximated, followed by the closure of the platysma, subcutaneous tissues, and skin after the placement of a drain.

## Results

A liquid diet was begun the day after surgery and was readily advanced. His surgical drain was removed the next day, and he was observed for an additional 24 hours. The patient was seen a week and then 10 weeks following surgery for routine postoperative follow‐up. Stroboscopy at 10 weeks revealed trace residual edema (2H), and baseline voice‐related quality of life measures. Postoperative recovery was remarkable only for 8 weeks of mild odynophagia managed with over‐the‐counter analgesics. The pathology returned as a 1.1 cm grade 1 chondrosarcoma with negative margins. He remained clear of disease at 18 months postoperatively.

## Discussion

Our team has previously described combining a transcervical open partial laryngectomy approach with the preservation of endolaryngeal soft tissues for a case of osteoblastoma of the thyroid cartilage.^5^ The current account demonstrates that a similar approach can be applied to low‐grade chondrosarcomas of the laryngeal framework, where endolaryngeal soft tissues do not need to be resected for disease control. This case also demonstrates that tracheostomy is not required if laryngeal mucosa is not violated. To our knowledge, these are the only such published reports.

Traditional open partial laryngectomy requires adequate cardiopulmonary reserve given the risk for postoperative aspiration, but in our technique, this is of less concern. Patients should be counseled about the risk of temporary postoperative edema or inadvertent mucosal violation and subcutaneous emphysema that may necessitate perioperative intubation or tracheostomy. While significant dysphagia is not anticipated, dissection of extrinsic muscular attachments to the larynx and postoperative swelling can cause temporary swallowing alterations. Finally, if this technique is used near the anterior commissure, there is a risk of dysphonia with disruption of the commissural attachments even with surgical resuspension of the anterior commissure tendon.

## Conclusion

Application of laryngeal framework surgical principles allows for a function‐sparing tailored open partial laryngectomy for select non‐squamous cell laryngeal pathologies which accomplishes full oncologic resections with no functional sequelae.

## Author Contributions

**Janice L. Farlow**, design, conduct, analysis, final approval of the manuscript; **Norman D. Hogikyan**, design, conduct, analysis, final approval of the manuscript; **Robert J. Morrison**, design, conduct, analysis, final approval of the manuscript.

## Disclosures

### Competing interests

None.

### Funding source

None.
